# Expanding the taxonomic and environmental extent of an underexplored carbon metabolism—oxalotrophy

**DOI:** 10.3389/fmicb.2023.1161937

**Published:** 2023-05-04

**Authors:** Alexander Sonke, Elizabeth Trembath-Reichert

**Affiliations:** School of Earth and Space Exploration, Arizona State University, Tempe, AZ, United States

**Keywords:** oxalate, oxalotrophy, carbon sequestration, oxalate-carbonate pathway, carbon cycle, biosignature

## Abstract

Oxalate serves various functions in the biological processes of plants, fungi, bacteria, and animals. It occurs naturally in the minerals weddellite and whewellite (calcium oxalates) or as oxalic acid. The environmental accumulation of oxalate is disproportionately low compared to the prevalence of highly productive oxalogens, namely plants. It is hypothesized that oxalotrophic microbes limit oxalate accumulation by degrading oxalate minerals to carbonates via an under-explored biogeochemical cycle known as the oxalate-carbonate pathway (OCP). Neither the diversity nor the ecology of oxalotrophic bacteria is fully understood. This research investigated the phylogenetic relationships of the bacterial genes *oxc*, *frc*, *oxdC*, and *oxlT*, which encode key enzymes for oxalotrophy, using bioinformatic approaches and publicly available omics datasets. Phylogenetic trees of *oxc* and *oxdC* genes demonstrated grouping by both source environment and taxonomy. All four trees included genes from metagenome-assembled genomes (MAGs) that contained novel lineages and environments for oxalotrophs. In particular, sequences of each gene were recovered from marine environments. These results were supported with marine transcriptome sequences and description of key amino acid residue conservation. Additionally, we investigated the theoretical energy yield from oxalotrophy across marine-relevant pressure and temperature conditions and found similar standard state Gibbs free energy to “low energy” marine sediment metabolisms, such as anaerobic oxidation of methane coupled to sulfate reduction. These findings suggest further need to understand the role of bacterial oxalotrophy in the OCP, particularly in marine environments, and its contribution to global carbon cycling.

## 1. Introduction

Plant production of oxalate biominerals appears to be widespread and oxalate minerals are stable over geologic time, yet oxalates are uncommon in the geologic record (Hofmann and Bernasconi, 1998; Stephens, 2012). Moreover, soil oxalate concentrations reported in the literature are often below detection limits, or on the order of a few micromolar—far less than would be expected given the apparent extent of production (Jones, 1998; Strobel, 2001). Limited accumulation appears to be due to degradation of oxalate minerals to carbonates by bacteria and fungi in an underexplored biogeochemical cycle known as the oxalate-carbonate pathway (OCP; Verrecchia, 1990). The global role of this pathway in carbon cycling is unconstrained but estimated to have significant influence on carbon fluxes and long-term sequestration. For example, deserts of the American Southwest and Mexico, where density of the oxalogenic Saguaro cactus is high, may accumulate as much as 1.8 × 10^11^ g yr.^−1^ of atmospheric carbon in oxalate biominerals (Garvie, 2006).

Oxalate occurs naturally as organic minerals and oxalic acid (Baran, 2014), forming by diagenesis, biomineralization, and abiotic processes such as radiolysis and ultraviolet (UV) irradiation (Vandenborre et al., 2021; Zhao et al., 2022). Calcium oxalate, in its monohydrate and dihydrate forms (known as whewellite and weddellite, respectively), is especially prevalent as a biomineral produced by plants, fungi, and lichens, and is present pathologically in animals (Bungartz et al., 2004; Franceschi and Nakata, 2005). In the plant kingdom, oxalogens are present in nearly 80% of families (McNair, 1932; Barth, 2020), and in some cases comprise up to 80% (w/w) of a plant’s dry weight and 90% of its total calcium (Nakata, 2003). Oxalates have been observed to serve biochemical, photosynthetic, and reproductive purposes in plants (He et al., 2014).

**Figure 1:**
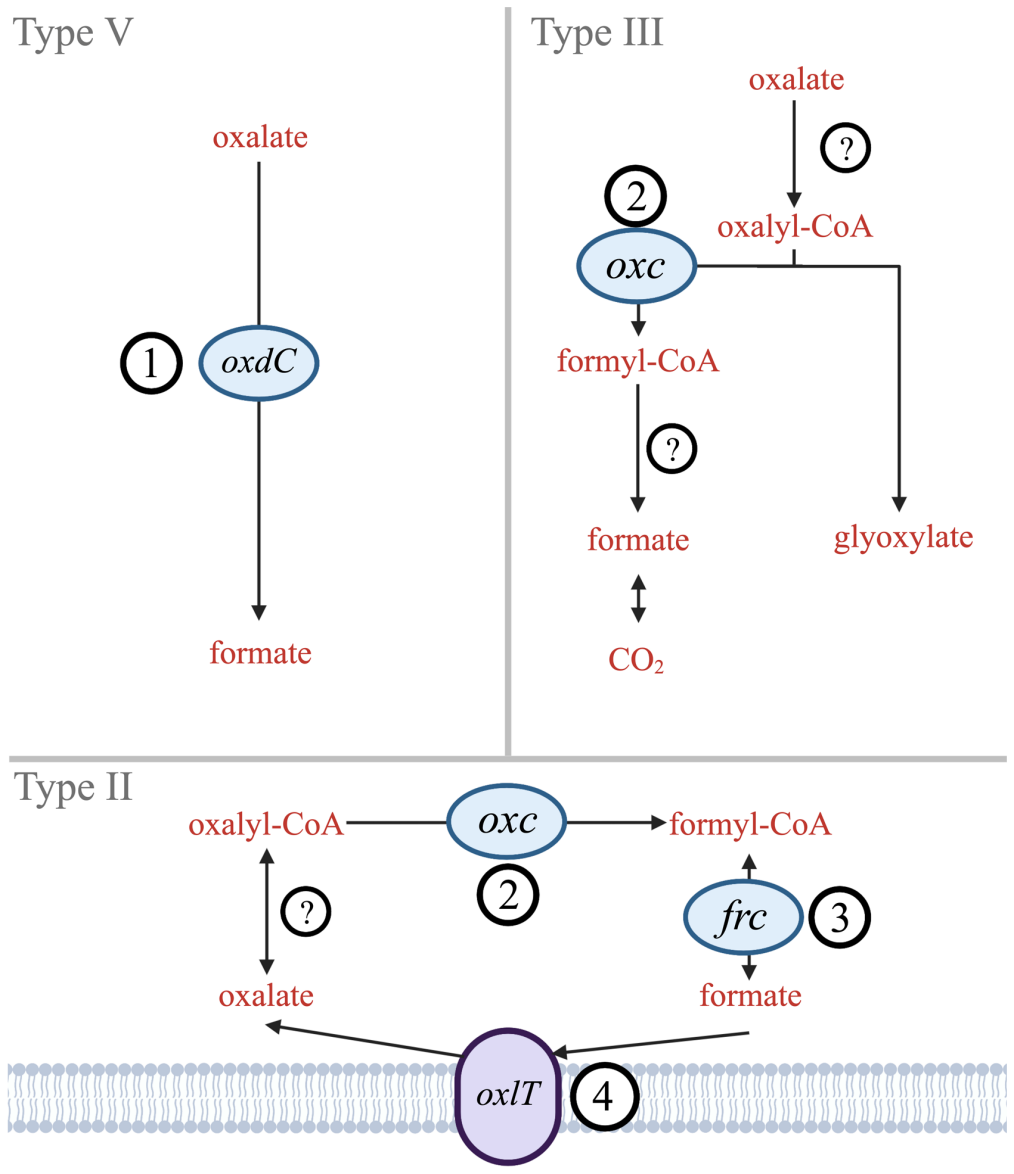
Simplified diagram of Type II, III, and V oxalate degradation pathways. Genes that encode key enzymes or proteins are labeled (1) *oxdC –* oxalate decarboxylase, (2) *oxc –* oxalyl-CoA decarboxylase, (3) *frc –* formyl-CoA transferase, and (4) *oxlT –* oxalate:formate antiporter. Hypothesized enzymes without known genes are indicated by (?) – oxalate CoA-transferase and formate-CoA hydrolase.

By contrast, bacterial oxalate use has centered around metabolic needs. Six biologically mediated oxalate degradation pathways are documented in MetaCyc (Caspi et al., 2018). Three are widespread in bacteria (Types II, III, V; Figure 1), while other types (I, IV, and VI) are either more common in eukaryotes (mostly plants) or specific bacteria (acetogens). Type V is a single-step pathway that utilizes the *oxdC* gene to directly convert oxalate to formate (Tanner and Bornemann, 2000). Types II and III are multistep pathways that convert oxalate to formate (Type II) or to CO_2_ (Type III). The key gene shared in both Type II and Type III pathways is *oxc*, which encodes oxalyl-CoA decarboxylase (Baetz and Allison, 1989; Lung et al., 1994). The Type II pathway also includes *frc*, which encodes formyl-CoA transferase (Baetz and Allison, 1990). While *frc* is well characterized, it is also ubiquitous across metabolisms and therefore not unique to oxalotrophy. Type II and III share EC 2.8.3.2 (oxalate CoA-transferase) as the initial catalyzing step in the pathway; however, no genes have been identified that encode this enzyme since its description in 1961 (Quayle et al., 1961). Similarly, Type III requires EC 3.1.2.10 (formyl-CoA hydrolase), which has not been associated with an encoding gene since its description in 1963 (Sly and Stadtman, 1963). The terminal step in Type III is formate dehydrogenase (Rusching et al., 1976). Types II, III, and V may all rely on an oxalate:formate antiporter, encoded by the *oxlT* gene. *oxlT* is thought to mediate transmembrane uptake of oxalate, which has been identified in fungi and many anaerobic bacteria, but only two strains of aerobic bacteria (Fu et al., 2001; Müller et al., 2016; Robertson and Meyers, 2022).

Therefore, this study investigated the taxonomic and environmental diversity of the key genes unique or essential to bacterial oxalotrophy: *oxdC* (1), *oxc* (2), *frc* (3), and *oxlT* (4) (Figure 1). Since presence of these genes in an organism guarantees neither metabolic function, nor microbial activity (Turroni et al., 2007; Bravo et al., 2013), we also provide transcript and protein amino acid conservation analysis. We investigated transcriptomes of contrasting ecosystems, evaluated the energetics of bacterial oxalotrophy, and compared conservation of key residues from characterized proteins across our gene alignments. In combination, these findings suggest novel taxa and environments where oxalotrophy may play a role in carbon cycling.

## 2. Materials and methods

### 2.1. Sequence data acquisition

Sequence data were obtained from NCBI using BLAST+ (v2.12.0; Altschul et al., 1997; Camacho et al., 2009). Each search consisted of FASTA-formatted amino acid sequences queried against nonredundant isolate and whole-genome sequencing (WGS) protein databases. Query sequences were obtained from reference oxalotrophic strains using their respective KEGG Orthology identifiers (KO IDs). Complexity of BLAST results was reduced to our target of fewer than 200 sequences by selecting for E-values less than 1e^−50^, percent identities below 0.87, and default BLAST+ parameters except where otherwise noted in Supplementary Table 1. Data processing was conducted with Unix commands in the MacOS Terminal bash shell.

Metatranscriptome searches were conducted using the IMG/MER OMICS/RefSeq database (Chen et al., 2023). Search queries were composed using the gene product name and the EC or KO ID listed in Table 1 to ensure accuracy. To evaluate the potential for active oxalotrophy in high temperature marine systems, we searched the one available hydrothermal RNA-seq dataset on IMG consisting of metatranscriptomes from hydrothermal plume and vent fluids of the Gulf of California and North Pacific Ocean (Anderson et al., 2019). From this, we recovered multiple genes related to oxalotrophy from plume samples, but only *frc* from vent fluids (Table 1). To compare with active oxalotrophy in a soil environment, we selected an available RNA-seq dataset looking at soil activation during spring snow melt (Brodie et al., 2017). From this we recovered high (>1,000 counts) transcription of all oxalotrophy genes of interest (Table 1). For comparison, we also provided the total gene count for each gene in the IMG isolate database.

**Table 1:** Transcript counts for genes involved in oxalotrophy from 36 soil and 3 hydrothermal vent fluid and 2 hydrothermal vent plume metatranscriptomes.

Gene	Gene name		EC number	KO ID	Soil MetaT (*n* = 36)	Vent MetaT (*n* = 3)	Plume MetaT (*n* = 2)	IMG isolate gene count
4	OFA family oxalate/formate antiporter-like MFS transporter	*oxlT*	–	K08177	3,178	0	2	38,263
3	Formyl-CoA transferase	*frc*	2.8.3.16	–	31,497	3	183	20,981
2	Oxalyl-CoA decarboxylase	*oxc*	4.1.1.8	–	2,452	0	13	9,997
1	Oxalate decarboxylase	*oxdC*	4.1.1.2	–	2,560	0	3	8,670

Sequences of fungal genes were obtained from FungiDB (Basenko et al., 2018) and included in the database prior to multiple sequence alignment in order to generate phylogenetic outgroups. In the case of *oxc*, the fungal sequences recovered represented predicted genes only. A neighbor-joining tree of all gene sequences used in this study suggested phylogenetic relationships that support the validity of using these fungal sequences, as well as those of the other genes investigated here, as outgroups (Supplementary Figure 1). Metadata for all sequences retained and used in this study are available in Supplementary Table 2.

### 2.2. Phylogenetic trees and sequence analysis

**Figure 2:**
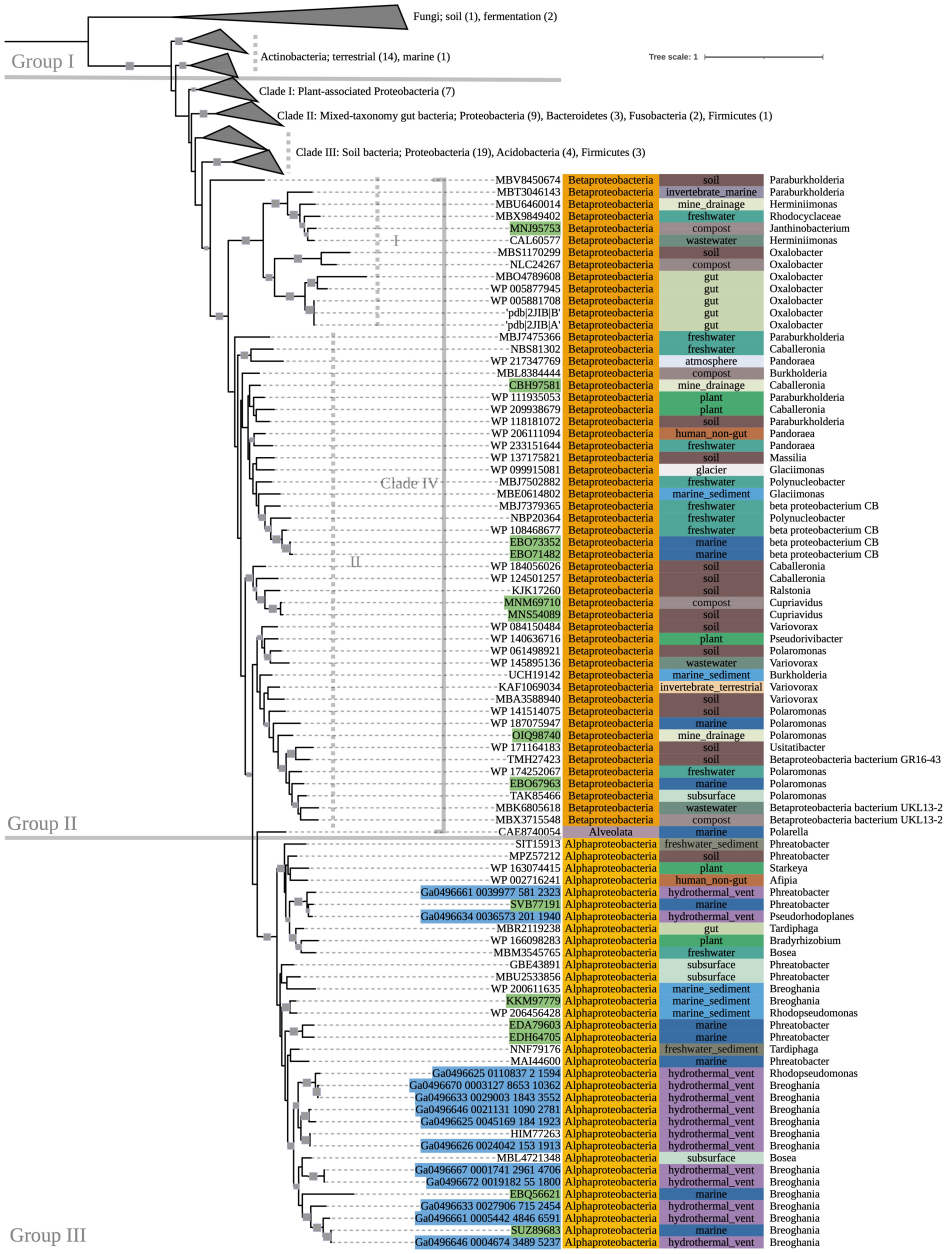
500-bootstrap maximum-likelihood phylogenetic tree of bacterial and predicted fungal *oxc* genes. Bootstrap values >50 displayed as gray boxes (min = 50, max = 100). Green-highlighted sequence identifiers indicate MAG genes. Blue-highlighted sequence identifiers indicate hydrothermal transcripts. Left-most annotation column indicates phylum taxonomy, class for Proteobacteria. Center column indicates source environment. Right-most column indicates genus taxonomy, or the next most exclusive classification available. Number of sequences included in collapsed nodes are indicated in parentheses.

Multiple-sequence alignment was performed with MUSCLE (v3.8.1551) using default parameters (Edgar, 2004). Sequence metadata, such as source environment and taxonomy, were obtained from multiple sources: NCBI archival protein sequence records, the JGI Integrated Microbial Genomes and Microbiomes, the BioCyc, BacDive, and Genomes OnLine databases (Karp et al., 2019; Reimer et al., 2022; Chen et al., 2023; Mukherjee et al., 2023), and directly from source publications. GhostKOALA (v2.2) was used to assign taxonomies of metagenome-assembled genome (MAG) sequences that were otherwise unclassified in NCBI, and to confirm gene annotations of all other sequences (Kanehisa et al., 2016). RAxML was used to construct maximumlikelihood phylogenetic trees with PROTCATAUTO model settings and branch support values generated from 500 bootstrapping iterations (Stamatakis, 2014). Trees were visualized and annotated using Interactive Tree of Life (iTOL v6.6; Letunic and Bork, 2021). Several nodes on the *oxc* phylogenetic tree (Figure 2), which was significantly larger than the other trees, were collapsed for simplification; the fully expanded version can be viewed in Supplementary Figure 4.

Multiple-sequence alignments of each gene were evaluated in Jalview (v2.11.2.5; Waterhouse et al., 2009) in order to assess key residue conservation, an indicator of the preservation of structure and function in the proteins encoded by each gene. This was then visualized by generating pore logos in WebLogo 3 (Schneider and Stephens, 1990; Crooks et al., 2004). Key residues were identified from experimental mutagenesis publications referenced in the Uniprot Knowledgebase (UniprotKB) (The UniProt Consortium, 2021). These publications show varying effects of mutation between residues. For simplicity, all referenced residues were considered “key” and assessed for conservation (see Results and Discussion).

### 2.3. Energetics

The Water-Organic-Rock-Microbe (WORM) Portal was used for all energetics calculations (Boyer et al., 2022). Code is provided in Supplementary Data Sheet 1.

## 3. Results

### 3.1. Phylogeny

The phylogeny of OXC-encoding sequences partitioned into groups (I, II, and III) generally attributable to sequence taxonomy at the phylum and class levels (Figure 2). Group I consisted entirely of Actinobacteria (bootstrap value = 100). Source environments for this group were predominantly terrestrial, with one marine exception collected from tissue of a coral reef-inhabiting sea sponge in the South China Sea (WP_111862994). Group II consisted of several Proteobacteria-dominated clades largely of terrestrial origin. Conversely, Group III was homogeneously comprised of Alphaproteobacteria from marine source environments. The marine hydrothermal plume transcriptome sequences included in this tree were predicted in GhostKOALA to be from Alphaproteobacteria, consistent with their placement in Group III among other sequences of marine and hydrothermal source environments.

Within Group II, Clade I contained a mixed group of Proteobacteria sequences that were documented to have been specifically isolated from plant hosts (as opposed to being labeled simply as “soil” isolates, for example). All Group II non-Proteobacteria sequences, including those that may represent previously undescribed oxalotrophic diversity (e.g., Fusobacteria, Deltaproteobacteria, Acidobacteria, and a single sequence purportedly isolated from the dinoflagellate *Polarella glacialis*) grouped into Clades II and III, along with Proteobacteria sequences of similar source environments. Clade II consisted predominantly of gut-associated sequences, while Clade III consisted of soil-associated sequences. Clade IV consisted of Betaproteobacteria sequences of mixed source environments that grouped into two subclades (Clade IV.I, Clade IV.II, bootstrap value = 91).

**Figure 3:**
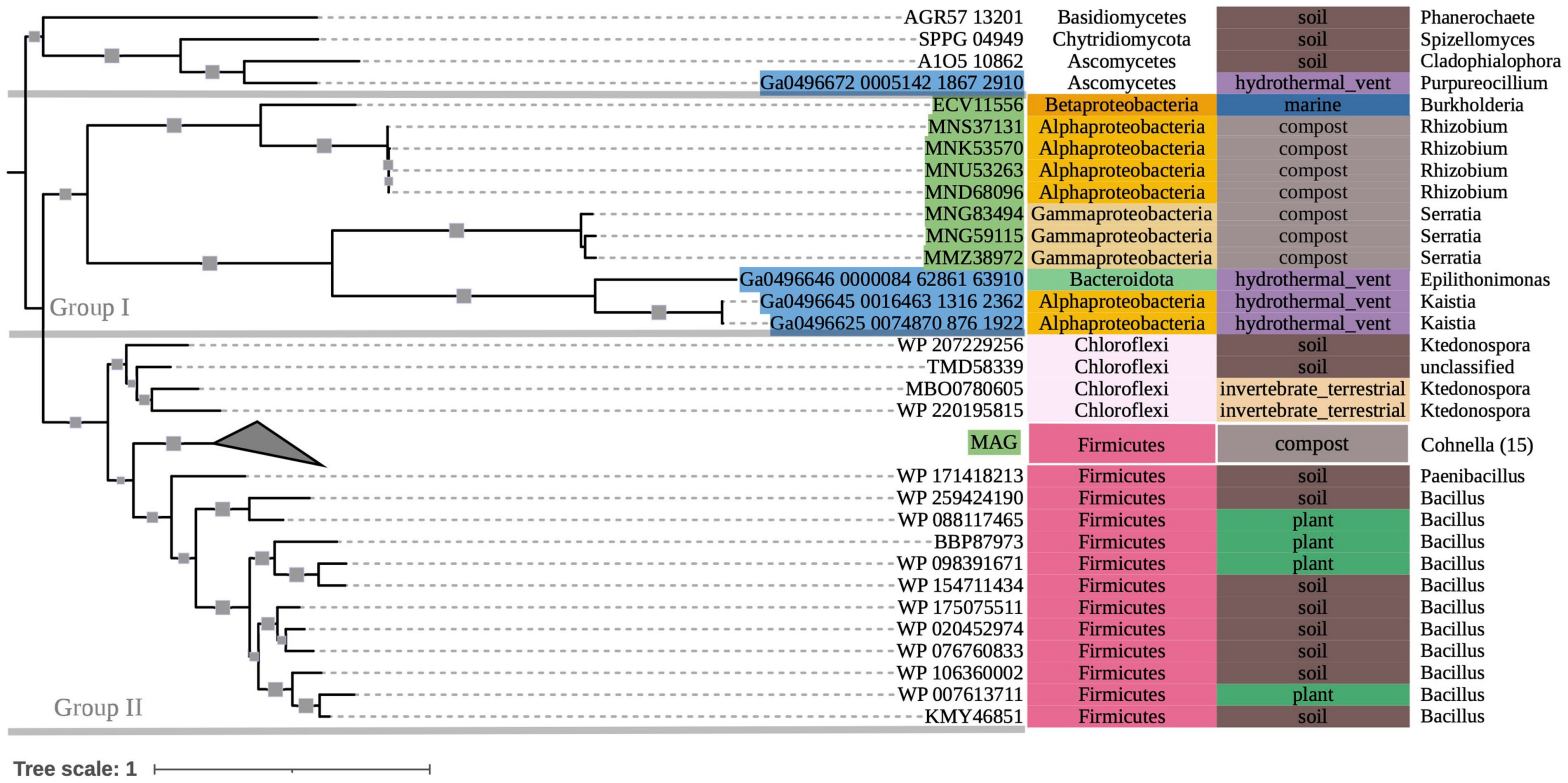
500-bootstrap maximum-likelihood phylogenetic tree of bacterial and fungal *oxdC* genes. Bootstrap values >50 displayed as gray boxes (min = 50, max = 100). Green-highlighted sequence identifiers indicate MAG genes. Blue-highlighted sequence identifiers indicate hydrothermal transcripts. Left-most annotation column indicates phylum taxonomy, class for Proteobacteria. Center column indicates source environment. Right-most column indicates genus taxonomy, or the next most exclusive classification available. Collapsed node represents 15 Firmicutes sequences from a single compost metagenome (NCBI BioProject PRJNA488358).

The phylogenetic tree of OXDC-encoding sequences (Figure 3) also demonstrated grouping by taxonomy. Group I consisted primarily of mixed Proteobacteria, while Group II consisted of Terrabacteria. Notably, Proteobacteria and Firmicutes (Terrabacteria) sequences that were obtained from the same compost metagenome sample grouped with their respective taxonomic groups rather than with each other. Taxonomic predictions in GhostKOALA assigned three of the included marine hydrothermal plume transcriptome sequences as bacterial (two Alphaproteobacteria and one Bacteroidetes), and one as fungal. The Bacteroidetes-assigned sequence grouped with the Alphaproteobacteria-assigned sequences among other Group I Proteobacteria of mixed source environments, while the fungal sequence grouped with the tree’s fungal outgroup.

**Figure 4:**
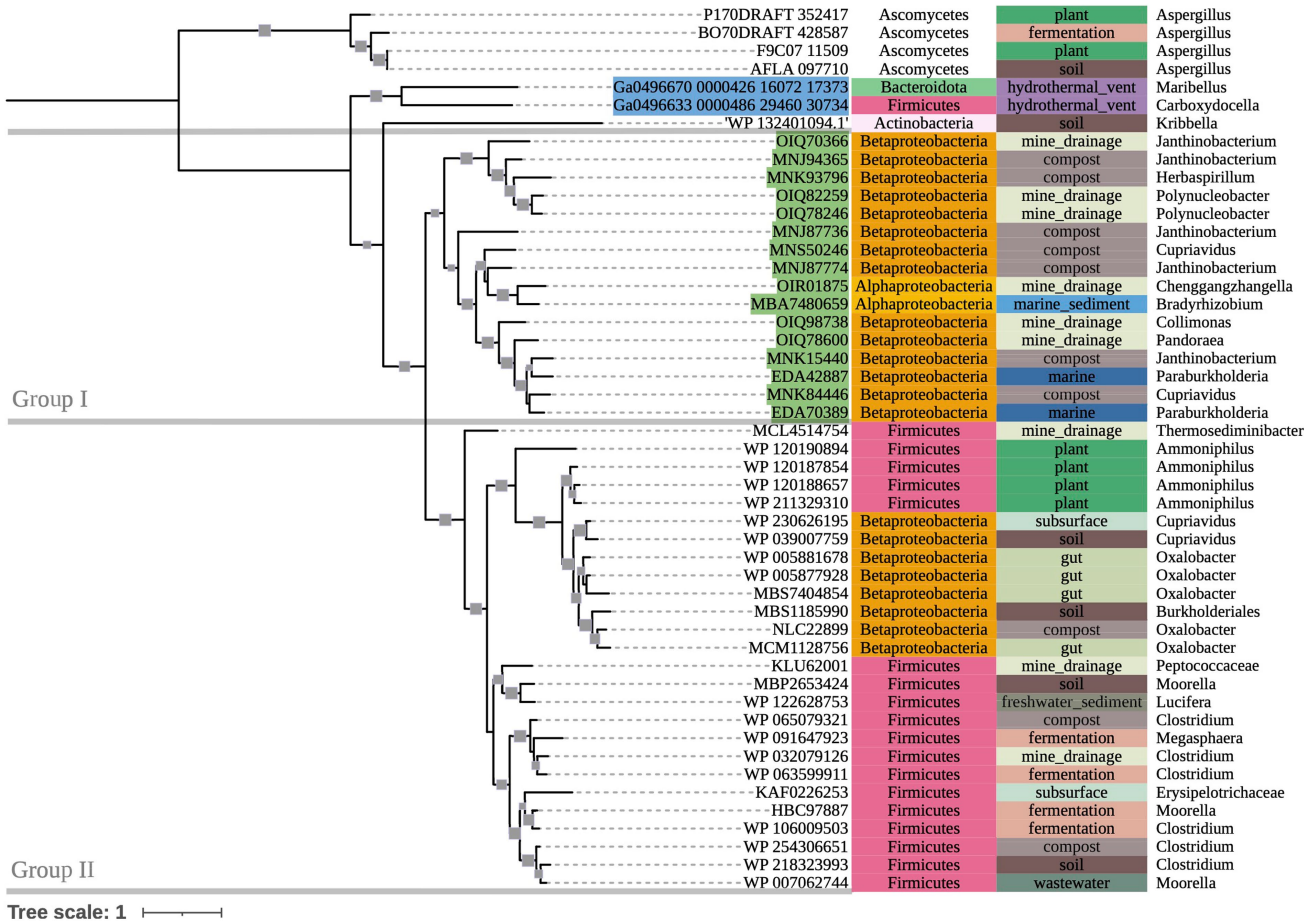
500-bootstrap maximum-likelihood phylogenetic tree of bacterial and fungal *oxlT* genes. Bootstrap values >50 displayed as gray boxes (min = 50, max = 100). Green-highlighted sequence identifiers indicate MAG genes. Blue-highlighted sequence identifiers indicate hydrothermal transcripts. Left-most annotation column indicates phylum taxonomy, class for Proteobacteria. Center column indicates source environment. Right-most column indicates genus taxonomy, or the next most exclusive classification available.

The phylogenetic tree of OXLT-encoding sequences (Figure 4) showed two main groups (bootstrap value = 90). Group I consisted of terrestrial-dominant Proteobacteria. Group II consisted mostly of terrestrial Firmicutes, with a single clade of terrestrial Betaproteobacteria within the group (bootstrap value = 100). Marine hydrothermal plume transcriptome sequences grouped together outside of Groups I and II (bootstrap value = 66), despite one of them appearing to share ancestry with sequences in Group II (Ga0496633_0000486_29460_30734).

The tree of *frc* sequences (Supplementary Figure 2) did not demonstrate distinct groupings. Rather, it showed numerous small clades of mixed taxonomic and environmental groups (bootstrap values ranged 1–80), which we attribute to the ubiquity and versatility of the formyl-CoA transferase enzyme. However, several sequences recovered from marine isolates and MAGs grouped with the hydrothermal vent transcriptome sequences included in our study.

### 3.2. Key residue conservation

**Figure 5:**
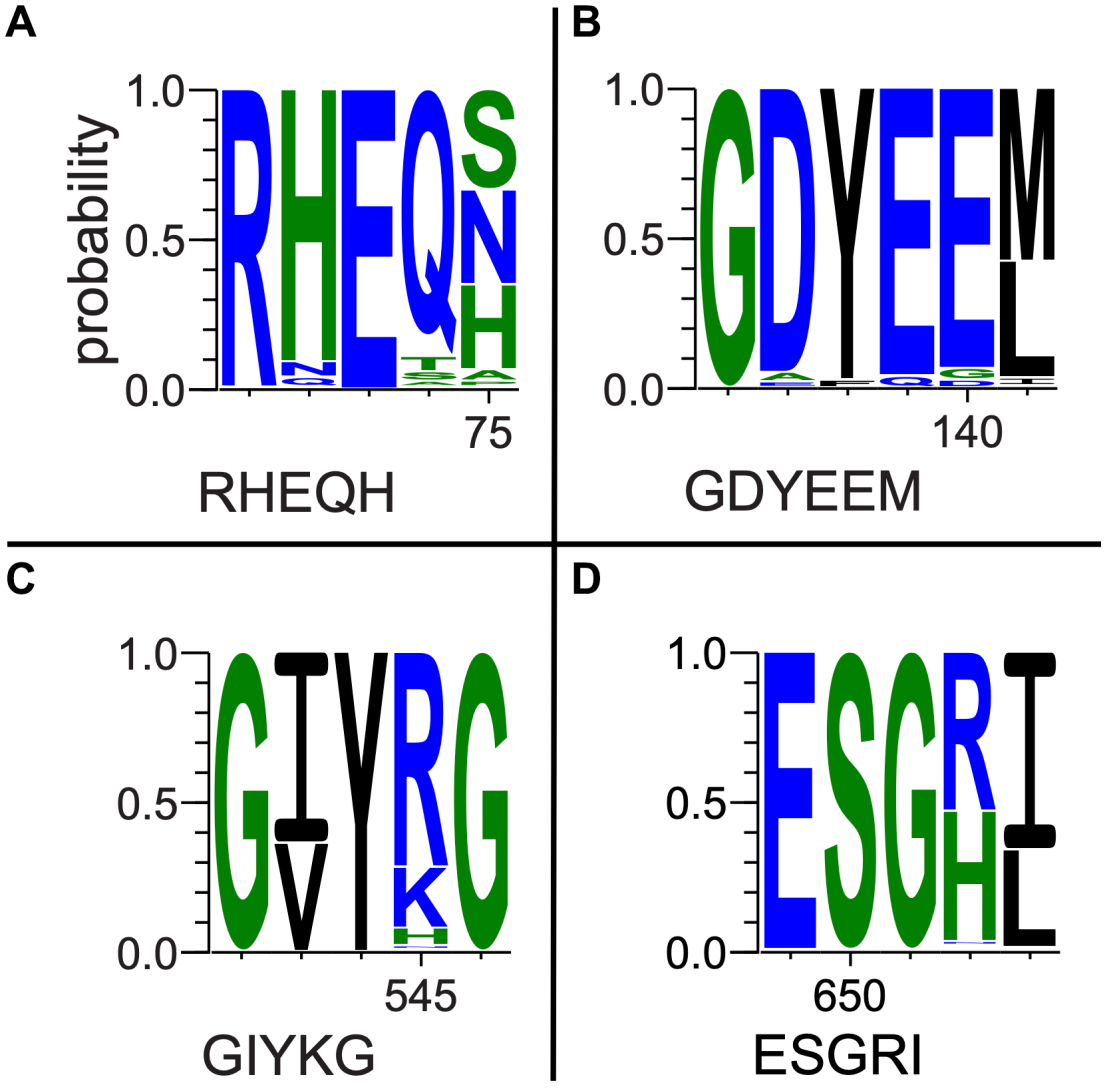
Selected oxc pore logos demonstrating conservation of **(A)** E-56, **(B)** Y-120, E-121, **(C)** Y-483, **(D)** S-553, and G-555 in a multiple-sequence alignment of bacterial isolate, MAG, and hydrothermal transcriptome genes and predicted fungal oxc genes. Letter strings below each logo represent partial reference gene sequences as documented in UniprotKB (The UniProt Consortium, 2021).

Multiple-sequence alignments that included fungal outgroups and hydrothermal transcripts were compared to each gene’s respective reference sequence in UniprotKB and assessed for key residue conservation. Key residues in the *oxc* gene (Figure 5 and Supplementary Figure 4) include E-56, Y-120, E-121, Y-483, S-553, and R-555 (Berthold et al., 2007). E-56 (Figure 5A) was conserved across all sequences (percentage identity; PID = 100%). Y-120 (Figure 5B) was conserved across all but two bacterial isolate sequences and two fungal sequences that all had phenylalanine substitutions (PID = 96.8%). E-121 (Figure 5B) was conserved across all but four bacterial isolate sequences and two fungal sequences that had glutamine substitutions (PID = 95.5%). Y-483 (Figure 5C) was conserved across all but three bacterial MAG sequences that terminated prior to this position (PID = 98.1%). Similarly, S-553 (Figure 5D) was conserved across all but eight bacterial sequences that terminated prior to said position and two fungal sequences which showed substitutions (PID = 93.5%). R-555 (Figure 5D) was conserved with only 50.3% consensus; many bacterial sequences either showed histidine substitution or terminated prior to this position.

Among a subset of 27 *oxc* genes of particular interest to this study, which include marine MAG sequences, hydrothermal transcripts, and sequences of taxa previously unknown to be oxalotrophic, key residue conservation was considerably high. E-56, Y-120, and E-121 were 100% conserved. Y–483 was conserved in all but three marine MAG sequences which terminated prior to this position (PID = 90.3%). S-553 was conserved in all but eight sequences (six MAGs, two hydrothermal transcripts; PID = 74.1% overall). R-555 showed similar conservation to the complete alignment, where terrestrial sequences had histidine substitutions, but marine sequences conserved arginine (74.1% occupancy, PID = 69.6% R).

Key residues in the *oxdC* gene (Supplementary Figure 4) include R-270, E-333, and Y-340 (Anand et al., 2002). R-270 was conserved across all sequences in our alignment (PID = 100%). E-333 was highly conserved among Terrabacteria sequences and fungi, but showed varying substitutions among nearly all non-Terrabacteria sequences (PID = 78%). Y-340 was highly conserved among non-Terrabacteria sequences, but showed frequent phenylalanine substitutions among Firmicutes and fungal sequences (PID = 52%). Our *oxdC* genes of interest (as detailed above) also had significant conservation of key residues. R-270 was completely conserved, while Y-340 had a single phenylalanine substitution in our hydrothermal fungal transcript (Ga0496672_0005142_1867_2910), consistent with our other fungal *oxdC* genes. E-333 was conserved among terrestrial sequences of interest (PID = 62.5%).

Key residues in the *frc* gene (Supplementary Figure 5) include Q-17, W-48, D-169, G-259, and G-260 (Toyota et al., 2008). Notably, all were completely conserved (PID = 100%) across all bacterial sequences in the alignment, with the exception of W-48 which was only present in seven of the sequences examined here (consensus Q 87.3%). Alternatively, fungal sequences had high conservation of D-240 only, and low conservation of the other key residues.

In the *oxlT* gene, 12 key residues are identified in UnitprotKB as having deleterious effects after mutagenic experimentation (Fu et al., 2001). Conservation of these residues ranged from 0 to 96%. However, a gene sequenced from *Kribbella albertanoniae* (the only Actinobacteria sequence on this tree), recently confirmed to be an aerobic oxalotroph that utilizes the OXLT enzyme (Bravo et al., 2013; Robertson and Meyers, 2022), showed conservation of only four of these 12 residues. Hence, we suspect that there may be greater variation in OXLT-encoding sequences than other key genes involved in oxalotrophy, and assessments of key residue conservation and subsequent conclusions about enzyme function in these organisms require more data of existing *oxlT* genotypes.

### 3.3. Energetics

The oxalotrophic reaction where bacteria catalyze the conversion of oxalate to calcite has been represented by Equation 1 (Garvie, 2006). This reaction represents the conversion of inorganic carbon into an organic component (represented by a generic CHO molecule) and inorganic bicarbonate formation in the presence of water. Based on known bacterial oxalotrophic catabolic pathways (Verrecchia et al., 2006), a more biologically relevant reaction would be oxalate converted to formate and bicarbonate. As a balanced overall reaction, this would be best represented by the oxalate ion and water converted to the formate ion and bicarbonate ion for circumneutral pH (Equation 2). Using Equation 2, we calculated the Gibbs free energy at standard state (ΔG_r_°) for a range of temperatures (2, 20, 50, 100°C) and pressures (1.01 and 250 bar) representative of marine and hydrothermal conditions which host other chemolithotrophic metabolisms (Nakamura and Takai, 2014) using the WORM portal (see Methods). These values ranged from −25.0 to −33.2 kJ/mol, with slightly higher yield at atmospheric pressures (Table 2). Temperature was a larger factor in ΔG_r_° than pressure, with the highest energy yield at the highest temperature (−33.1 and − 33.2 kJ/mol at 1.01 and 250 bar, respectively). For comparison, the ΔG_r_° of anaerobic oxidation of methane coupled to sulfate reduction (AOM-SR, Equation 3) ranges from 30.1 to −43.1 across the sample pressure/temperature space (Nakamura and Takai, 2014).

**Table 2:** Theoretical standard state Gibbs free energy (ΔG_r_°) for oxalotrophy (Equation 2) and anaerobic methanotrophy (Equation 3) across relevant marine temperatures and pressures.

Temperature (°C)	Pressure (bar)	EQ 2 ΔG_r_° (kJ/mol)	EQ 3 ΔG_r_° (kJ/mol)
2	1.01	−25.0	−30.6
20	1.01	−26.2	−33.0
50	1.01	−28.6	−36.9
100	1.01	−33.2	−43.1
2	250	−25.0	−30.1
20	250	−26.2	−32.6
50	250	−28.5	−36.6
100	250	−33.1	−43.0

While there is a paucity of data regarding the concentrations of oxalate in hydrothermal fluids and plumes, we can approximate significant ranges for concentrations that would affect the favorability of Equation 2 using the WORM portal. First, we assumed an activity of 1 M, 10 μM, and 2 mM for water, formate, and bicarbonate, respectively. Then we evaluated a range of activities for oxalate. For 100°C, activity of oxalate below 0.1 μM (10^−7^ M) reduced the favorability of (increased) ΔG_r_ and activities of 1 μM (10^−6^ M) or 10 μM (10^−5^ M) increased the favorability (lowered) ΔG_r_ to 38.8 kJ/mol and − 41.1 kJ/mol, respectively.


(1) $$C_{2}O_{4}^{2-}+H_{2}O+H^{+}\to HCO+HCO_{3}^{-}$$

(2) $$C_{2}O_{4}^{2-}+H_{2}O\to HCOO^{-}+HCO_{3}^{-}$$

(3) $$SO_{4}^{2-}+CH_{4}\to HS^{-}+HCO_{3}^{-}+H_{2}O$$

## 4. Discussion

Phylogenies of *oxc* and *oxdC* genes demonstrated grouping primarily by taxonomy. For example, *oxdC* clades of Proteobacteria and Terrabacteria from the same metagenomic compost sample grouped with their respective taxonomic groups rather than with each other, suggesting that their genes were inherited vertically (ancestrally) rather than by horizontal gene transfer (HGT). Although, some mixed-taxonomy clades (e.g., *oxc* Group II: Clades II and III)—including taxa not previously described as oxalotrophic—grouped by source environments instead of taxonomy, particularly from soil and gut microbiomes, where HGT is known to be prevalent (Heuer and Smalla, 2007; Berthold et al., 2016). HGT may still be a mechanism for gene proliferation among close relatives in these environments. As more oxalotrophic organisms are described from these novel environments, we hope to gain more resolution on these gene histories.

There may also be evidence for phylogenetic differences between *oxc* genes used in either predominantly anaerobic or aerobic oxalate degradation pathways (II and III, respectively; Figure 1). In Group II, some clades’ (Clade II, Clade IV-I) source environments were possibly anoxic, while others (Clade III, Clade IV-II) appear to range between hypoxic and highly oxygenated. This possible distinction between oxalate degradation pathways may also explain some grouping on our *oxlT* tree, which placed a clade of Betaproteobacteria sequences from anaerobe-hosting source environments (gut microbiomes, anaerobic digesters, etc.) in Group II (Firmicutes of mixed terrestrial environments) rather than Group I (other terrestrial-dominant Proteobacteria). Further sequencing of oxalotrophs isolated under varying conditions may help to better characterize the phylogenetics of oxalate degradation pathways.

While the conservation of protein activity in the novel sequences recovered by bioinformatic approaches requires further research, the highly conserved key residues in our *oxc* and *frc* alignments supports the potential for similar function across the alignments. Moreover, conservation among predicted fungal *oxc* genes supports their annotation as OXC-encoding sequences. Most notably, marine MAG sequences, hydrothermal transcripts, and sequences from taxa not previously known to be oxalotrophic had near complete conservation of key residues, supporting the possibility of unexplored oxalotrophy in their respective ecosystems and taxonomic groups. Where some metagenomic sequences of interest did not show key residue conservation due to termination, we question if they may be incomplete assemblies. Key residue conservation was interpreted to be less significant in our assessment of our *oxlT* alignment considering high variability and poor conservation in *Kribbella albertanoniae*, which is known to actively utilize the oxalate:formate antiporter (see Results). Similarly, our *oxdC* alignment, which used a reference gene sequenced from *Bacillus subtilis* (a Terrabacterium), showed poor conservation among non-Terrabacteria sequences. In both cases, this may suggest that amino acid sequences for these genes vary too widely to be recovered with only a single reference sequence query.

The known diversity of oxalotrophic bacteria was limited to the phyla Proteobacteria, Actinobacteria, and Firmicutes until 2017 with the discovery of oxalotrophic Bacteroidetes in a mouse gut microbiome (Tanca et al., 2017). Furthermore, active oxalotrophic bacteria have only been documented in terrestrial ecosystems (soil, freshwater sediments, plants, gut microbiomes and other host-associations, etc.). This study recovered gene sequences from public datasets that were taxonomically assigned to Fusobacteria, Deltaproteobacteria, Chloroflexi, and Acidobacteria, all of which may represent additional oxalotrophic groups not previously described in the literature. The purported *oxc* sequence from a marine isolate of *Polarella glacialis* (CAE8740054), a species known for its relatively large genome (billions of base pairs; Stephens et al., 2020) may represent the discovery of an oxalotrophic protist, but verification of transcription or metabolic activity would be required to determine if the gene is functional or simply non-encoding “junk” DNA.

Isolates, MAGs, and transcriptome sequences were also recovered in this study from diverse marine environments. These sequences were sampled from ocean and arctic surface water, oxygen minimum mesopelagic water, and sediments and diffuse fluids around hydrothermal vents (Table 1 and Supplementary Table 2). Oxalate is produced in such environments by algae (Pueschel, 2019) and porifera (Cerrano et al., 1999), as well as abiotic processes (Vandenborre et al., 2021; Zhao et al., 2022). Our energy calculations for oxalotrophy have a theoretical ΔG_r_° similar to low energy metabolisms such as AOM-SR (Nakamura and Takai, 2014), but are still energy-yielding. Therefore, oxalotrophy is plausible in marine systems, especially where hydrothermal fluids may provide both a source of oxalate and warmer temperatures. Overall, hot desert and tropical soils with oxalate sourced from plants may be the most energy-yielding environment for this metabolism.

## 5. Conclusion

**Figure 6:**
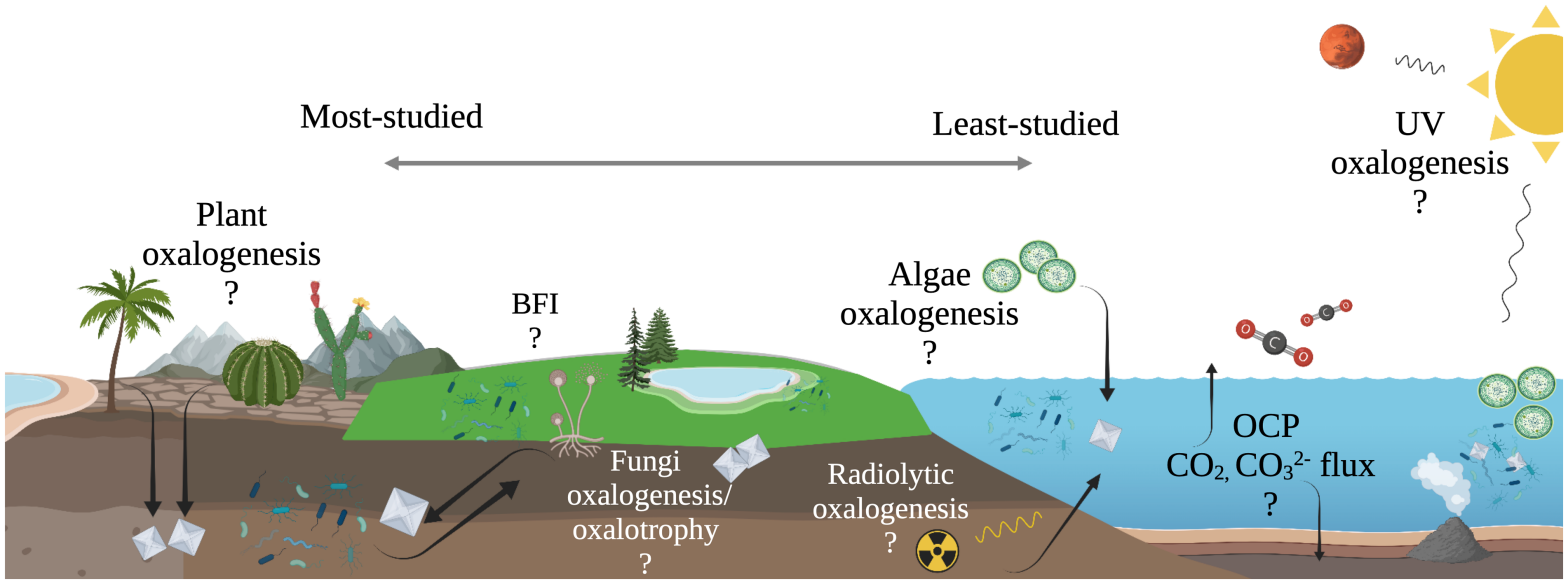
Illustration of unconstrained oxalate flux in the oxalate-carbonate pathway (OCP) and other possible, yet unexplored sub-cycles of global carbon cycling. Processes to the left are well-documented, while processes to the right are relatively unexplored.

The work presented here suggests the diversity of microbial oxalotrophs may be more extensive than previously recognized and oxalotrophs may occupy a significant niche in marine ecosystems, where oxalate sources are present but unconstrained (Figure 6). Furthermore, the abiotic processes that produce oxalate on Earth, and beyond, make oxalotrophy a candidate for astrobiological research. Oxalate minerals are thought to exist in a large, stable reservoir on Mars (Applin et al., 2015), and are known to be delivered to the inner solar system in meteorites (Peltzer et al., 1984; Shimoyama and Shigematsu, 1994). Continued study of the microbe-mineral interactions in the OCP may further elucidate their role in marine ecosystems, and thereby its role in global carbon cycling and as a potential signature of life in the universe.

## Data availability statement

The original contributions presented in the study are included in the article/Supplementary material, further inquiries can be directed to the corresponding author.

## Author contributions

AS and ET-R contributed to the conception and design of the study and read and approved the submitted version. AS developed the database, conducted the analyses, and wrote the first draft of the manuscript. ET-R contributed to the manuscript scope and revision. All authors contributed to the article and approved the submitted version.

## Conflict of interest

The authors declare that the research was conducted in the absence of any commercial or financial relationships that could be construed as a potential conflict of interest.

## Publisher’s note

All claims expressed in this article are solely those of the authors and do not necessarily represent those of their affiliated organizations, or those of the publisher, the editors and the reviewers. Any product that may be evaluated in this article, or claim that may be made by its manufacturer, is not guaranteed or endorsed by the publisher.
