# Supplementary material for: Expanding the taxonomic and environmental extent of an underexplored carbon metabolism—oxalotrophy
Source: Front Microbiol. 2023 May 4;14:1161937. doi: 10.3389/fmicb.2023.1161937 (PMC10192776; doi:10.3389/fmicb.2023.1161937)
Supplement: Supplementary file 1 [file Table_1.DOCX]

**Supplementary Table 1**. Selection parameters and number of sequences retained of each gene.

| Gene | NCBI Database | E-value | Sequence Length (AA) | CD-HIT Percent Identity threshold (%) | Sequences retained (no.) |
| --- | --- | --- | --- | --- | --- |
| oxc | nr | 0 | 400 | 87 | 123 |
|  | env_nr | 0 | 400 | 87 | 14 |
| frc | nr | 0 | 350 | 90 | 90 |
|  | env_nr | 0 | 350 | 90 | 22 |
| oxdC | nr | 0 | 300 | 90 | 16 |
|  | env_nr | 1e^-50^ | 300 | 90 | 23 |
| oxlT | nr | 1e^-100^ | 200 | 90 | 26 |
|  | env_nr | 1e^-50^ | 200 | 90 | 16 |
