# Supplementary material for: Expanding the taxonomic and environmental extent of an underexplored carbon metabolism—oxalotrophy
Source: Front Microbiol. 2023 May 4;14:1161937. doi: 10.3389/fmicb.2023.1161937 (PMC10192776; doi:10.3389/fmicb.2023.1161937)
Supplement: Supplementary file 3 [file Data_Sheet_1.PDF]

In [1]:

```
import pandas as pd
import AqEquil
ae = AqEquil.AqEquil()
#ae.half_cell_reactions
```

In [4]:

```
from pyCHNOSZ import *
basis(["H2O", "H+", "H2"])
results = subcrt(["CH4", "SO4-2","HS-","H2O", "HCO3-"],
                 [-1, -1, 1, 1, 1],
                 T=[2, 20, 50, 100],
                 P=[1.01325,1.01325,1.01325,1.01325,250, 250, 250, 250])
```

```
info.character: found CH4(aq); also available in gas, liq

info.character: found H2O(liq) [water]; also available in cr

subcrt: 5 species at 8 values of T (ºC) and P (bar) (wet) [energy units: cal]
```

| | coeff | name | formula | state | ispecies |
|---|---|---|---|---|---|
| **880** | -1 | CH4 | CH4 | aq | 880.0 |
| **24** | -1 | SO4-2 | SO4-2 | aq | 24.0 |
| **22** | 1 | HS- | HS- | aq | 22.0 |
| **1** | 1 | water | H2O | liq | 1.0 |
| **13** | 1 | HCO3- | HCO3- | aq | 13.0 |

| | T | P | rho | logK | G | H | S | V | |
|---|---|---|---|---|---|---|---|---|---|
| **1** | 2.0 | 1.01325 | 0.999950 | 5.813998 | -7319.840634 | 1408.949826 | 31.543122 | 19.799024 | -5.3 |
| **2** | 20.0 | 1.01325 | 0.998233 | 5.877750 | -7884.211475 | 1298.822333 | 31.155836 | 14.259372 | -6.6 |
| **3** | 50.0 | 1.01325 | 0.988030 | 5.957275 | -8808.645014 | 1088.807106 | 30.474243 | 9.857681 | -7.2 |
| **4** | 100.0 | 1.01325 | 0.958393 | 6.035623 | -10305.351861 | 721.719779 | 29.418120 | 7.027562 | -7.[illegible] |
| **5** | 2.0 | 250.00000 | 1.012082 | 5.718376 | -7199.452843 | 2307.584065 | 34.371567 | 20.631574 | -5.8 |
| **6** | 20.0 | 250.00000 | 1.009320 | 5.815280 | -7800.416299 | 1809.318241 | 32.611408 | 13.909175 | -6.[illegible] |
| **7** | 50.0 | 250.00000 | 0.998602 | 5.920369 | -8754.073482 | 1350.719249 | 31.115867 | 8.519580 | -7.4 |
| **8** | 100.0 | 250.00000 | 0.969682 | 6.014915 | -10269.994107 | 835.423180 | 29.628077 | 4.916000 | -7.5 |

In [77]:

```
from pyCHNOSZ import *
basis(["H2O", "H+", "H2"])
results = subcrt(["H2", "CO2","CH4", "H2O"],
                 [-1, -1, 1, 1],
                 T=[2, 20, 50, 100],
                 P=[1,1,1,1,250, 250, 250, 250])
```

```
info.character: found H2O(liq) [water]; also available in cr
```

```
subcrt: 4 species at 8 values of T (ºC) and P (bar) (wet) [energy units: cal]
```

| | coeff | name | formula | state | ispecies |
|---|---|---|---|---|---|
| **1102** | -1 | oxalate-2 | C2O4-2 | aq | 1102.0 |
| **1** | -1 | water | H2O | liq | 1.0 |
| **1077** | 1 | formate | HCO2- | aq | 1077.0 |
| **13** | 1 | HCO3- | HCO3- | aq | 13.0 |

| | T | P | rho | logK | G | H | S | V | Cp |
|---|---|---|---|---|---|---|---|---|---|
| **1** | 2.0 | 1 | 0.999949 | 4.749265 | -5979.339416 | -1789.216358 | 15.074695 | 1.846883 | 34.075409 |
| **2** | 20.0 | 1 | 0.998232 | 4.673950 | -6269.475212 | -1212.873678 | 17.104830 | 2.426309 | 30.573400 |
| **3** | 50.0 | 1 | 0.988030 | 4.617069 | -6826.966468 | -324.083914 | 19.992455 | 2.932203 | 29.163305 |
| **4** | 100.0 | 1 | 0.000590 | NaN | NaN | NaN | NaN | NaN | NaN |
| **5** | 2.0 | 250 | 1.012082 | 4.737102 | -5964.025971 | -1831.422030 | 14.865649 | 3.260980 | 33.929395 |
| **6** | 20.0 | 250 | 1.009320 | 4.660477 | -6251.403269 | -1227.805941 | 16.992245 | 3.615006 | 30.532412 |
| **7** | 50.0 | 250 | 0.998602 | 4.603229 | -6806.501889 | -322.561355 | 19.933838 | 3.919350 | 29.080187 |
| **8** | 100.0 | 250 | 0.969682 | 4.633469 | -7911.284566 | 1148.029144 | 24.164526 | 4.253685 | 29.640297 |

```
'below minimum density for applicability of revised HKF equations (1 T,P pair)'
```

In [5]:

```
from pyCHNOSZ import *
basis(["H2O", "H+", "O2"])
results = subcrt(["oxalate-2","H2O","formate","HCO3-"],
                 [-1, -1, 1, 1],
                 T=[2, 20, 50, 100],
                 P=[1.01325,1.01325,1.01325,1.01325,250, 250, 250, 250])
```

```
info.character: found H2O(liq) [water]; also available in cr

subcrt: 4 species at 8 values of T (ºC) and P (bar) (wet) [energy units: cal]
```

| | coeff | name | formula | state | ispecies |
|---|---|---|---|---|---|
| **1102** | -1 | oxalate-2 | C2O4-2 | aq | 1102.0 |
| **1** | -1 | water | H2O | liq | 1.0 |
| **1077** | 1 | formate | HCO2- | aq | 1077.0 |
| **13** | 1 | HCO3- | HCO3- | aq | 13.0 |

| | T | P | rho | logK | G | H | S | V | |
|---|---|---|---|---|---|---|---|---|---|
| **1** | 2.0 | 1.01325 | 0.999950 | 4.749265 | -5979.338831 | -1789.219590 | 15.074681 | 1.846965 | 34.0 |
| **2** | 20.0 | 1.01325 | 0.998233 | 4.673949 | -6269.474444 | -1212.875112 | 17.104822 | 2.426378 | 30.5 |
| **3** | 50.0 | 1.01325 | 0.988030 | 4.617068 | -6826.965540 | -324.084257 | 19.992451 | 2.932260 | 29.1 |
| **4** | 100.0 | 1.01325 | 0.958393 | 4.646963 | -7934.323544 | 1145.869058 | 24.220480 | 3.471028 | 29.9 |
| **5** | 2.0 | 250.00000 | 1.012082 | 4.737102 | -5964.025971 | -1831.422030 | 14.865649 | 3.260980 | 33.9 |

| | T | P | rho | logK | G | H | S | V |
|---|---|---|---|---|---|---|---|---|
| **6** | 20.0 | 250.00000 | 1.009320 | 4.660477 | -6251.403269 | -1227.805941 | 16.992245 | 3.615006 | 30.5 |
| **7** | 50.0 | 250.00000 | 0.998602 | 4.603229 | -6806.501889 | -322.561355 | 19.933838 | 3.919350 | 29.0 |
| **8** | 100.0 | 250.00000 | 0.969682 | 4.633469 | -7911.284566 | 1148.029144 | 24.164526 | 4.253685 | 29.6 |

In [38]:

```
#speciation = ae.speciate(input_filename="EPR_segE_vonDamm_2000.csv", verbose=0)
print("Done!")
```

```
Done!
```

In [61]:

```
#speciation.plot_mass_contribution("HCO3-")
```

In [62]:

```
#speciation.plot_mineral_saturation('EPR 9.6 N', plot_width=6)
```

In [6]:

```
import sympy as sym
import math
```

In [64]:

```
aC2O4 = 10**-5 # C2O4-2 activity
aH2O = 1 # H2O activity
aformate = 10**-5 # O2 activity
aHCO3 = 2*10**-3 # HCO3- activity
```

In [65]:

```
a1, a2, a3, a4 = sym.symbols('a_{C_{2}O_{4}^{2-}} a_{H_{2}O} a_{formate} a_{HCO_
Q = ((a1)*(a2))/((a3)*(a4))
Q
```

Out[65]:

$$\frac{a_{C_2O_4^{2-}} a_{H_2O}}{a_{HCO_3^-} a_{formate}}$$

In [66]:

```
Q_sub = Q.subs({"a_{C_{2}O_{4}^{2-}}": aC2O4,
                "a_{H_{2}O}":aH2O,
                "a_{formate}": aformate,
                "a_{HCO_{3}^{-}}": aHCO3
               })
```

In [67]:

```
float(Q_sub) # Q
```

Out[67]:

```
499.99999999999994
```

In [70]:

```
R=1.9872 #cal/K*mol
T_C=100
T_K=T_C+(-273.15)
lnQ=float(sym.ln(Q_sub)) # lnQ
(R*T_K*lnQ)*(4.184/1000) #kJ/mol
```

Out[70]: -8.946836418316014

In [ ]:
