# Supplementary material for: Expanding the taxonomic and environmental extent of an underexplored carbon metabolism—oxalotrophy
Source: Front Microbiol. 2023 May 4;14:1161937. doi: 10.3389/fmicb.2023.1161937 (PMC10192776; doi:10.3389/fmicb.2023.1161937)
Supplement: Supplementary file 4 [file Data_Sheet_2.docx]

**Supplementary Table 1**. Selection parameters and number of sequences retained of each gene.

**Supplementary Figure 1**. Unrooted neighbor-joining tree of bacterial and fungal *oxc, frc, oxdC, and oxlT* genes.

**Supplementary Table 2**. Sequence metadata for all genes used in this study.

**Supplementary Figure 2**. Uncollapsed 500-bootstrap maximum-likelihood phylogenetic tree of bacterial and predicted fungal *oxc* genes. Bootstrap values >50 displayed as gray boxes (min=50, max=100). Green-highlighted sequence identifiers indicate MAG genes. Blue-highlighted sequence identifiers indicate hydrothermal transcripts. Left-most annotation column indicates phylum taxonomy, class for Proteobacteria. Center column indicates source environment. Right-most column indicates genus taxonomy, or the next most exclusive classification available.

**Supplementary Figure 3**. 500-bootstrap maximum-likelihood phylogenetic tree of bacterial and fungal *frc* genes. Bootstrap values displayed as gray boxes (min=50, max=100). Green-highlighted sequence identifiers indicate MAG genes. Blue-highlighted sequence identifiers indicate hydrothermal transcripts. Left-most annotation column indicates phylum taxonomy, class for Proteobacteria. Center column indicates source environment. Right-most column indicates genus taxonomy, or the next most exclusive classification available.

**Supplementary Figure 4**. Multiple-sequence alignment pore logo of all *oxc* genes in Supplementary Table 2.

**Supplementary Figure 5**. Multiple-sequence alignment pore logo of all *oxdC* genes in Supplementary Table 2.

**Supplementary Figure 6**. Multiple-sequence alignment pore logo of all *frc* genes in Supplementary Table 2.

**Supplementary Figure 7**. Multiple-sequence alignment pore logo of all *oxlT* genes in Supplementary Table 2.

**Supplementary Datatset 1**. WORM portal Python notebook of oxalotrophy energetics calculations across pressure-temperature regimes.
