# Supplementary figures and images for: Expanding the taxonomic and environmental extent of an underexplored carbon metabolism—oxalotrophy
Source: Front Microbiol. 2023 May 4;14:1161937. doi: 10.3389/fmicb.2023.1161937 (PMC10192776; doi:10.3389/fmicb.2023.1161937)

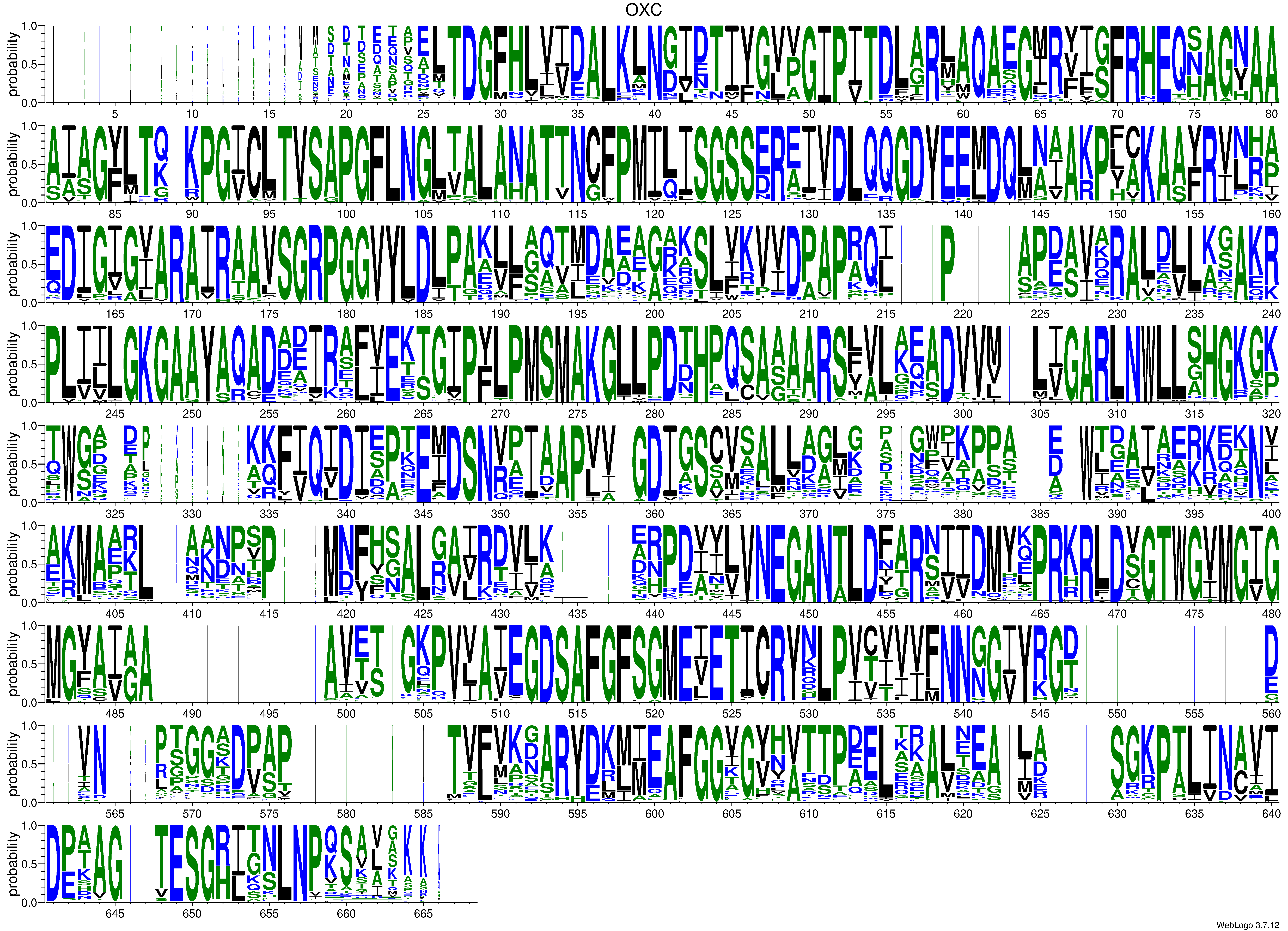

Supplement: Supplementary file 8 [file Image_4.PNG]

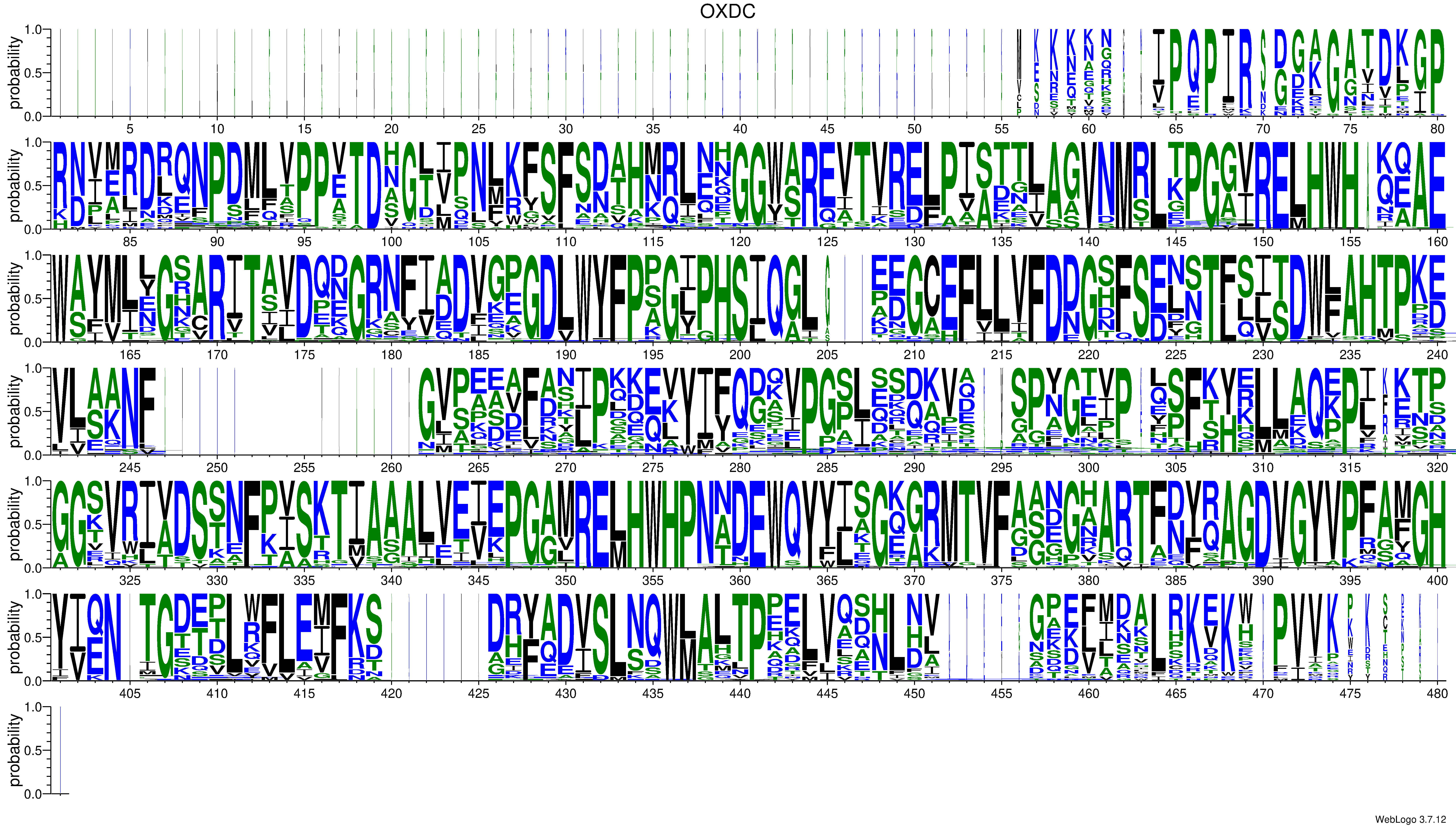

Supplement: Supplementary file 9 [file Image_5.PNG]

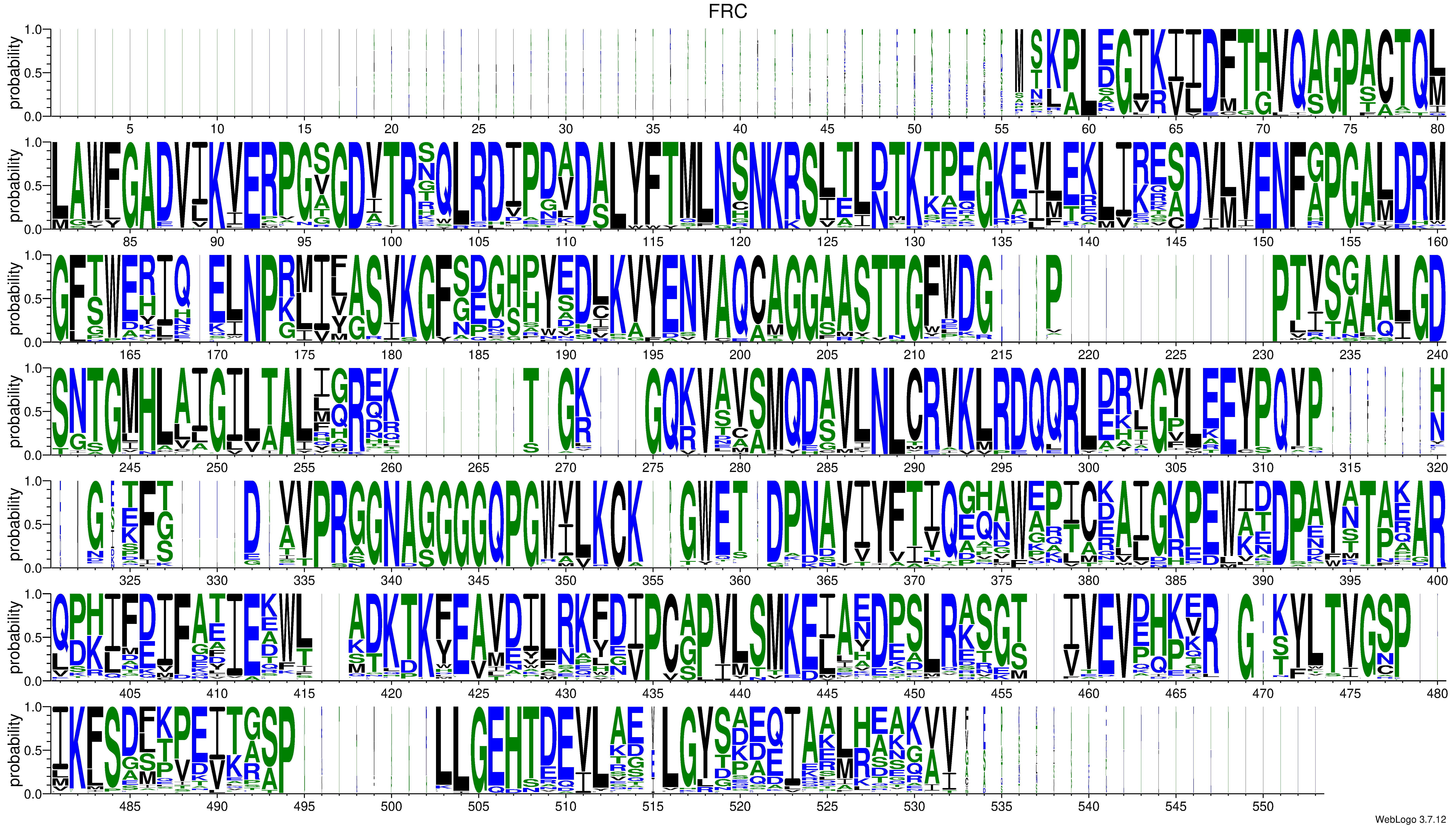

Supplement: Supplementary file 10 [file Image_6.PNG]

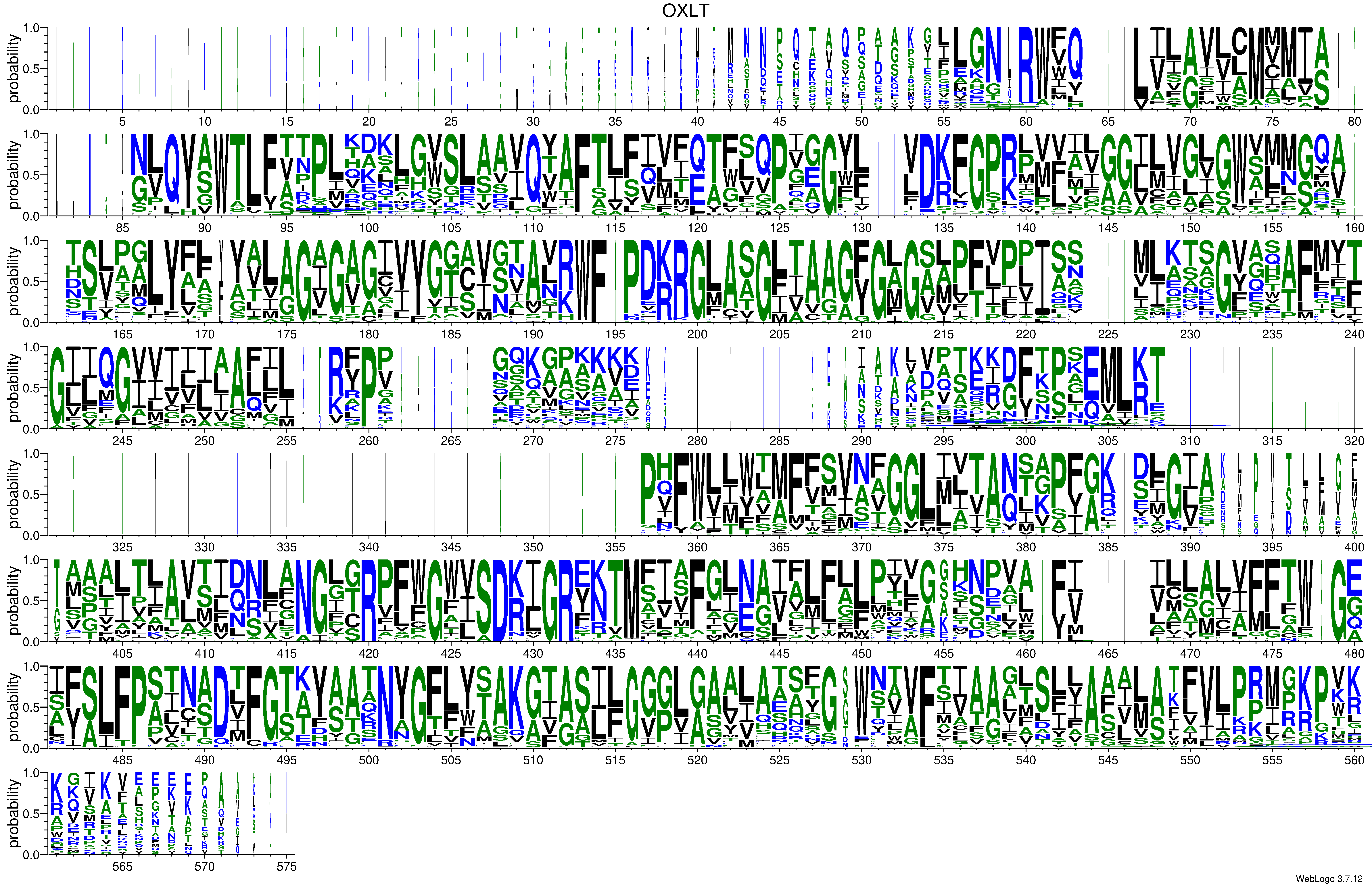

Supplement: Supplementary file 11 [file Image_7.PNG]
